# Optical characterisation of holographic diffusers and Bangerter foils for treatment of amblyopia

**DOI:** 10.1364/BOE.489585

**Published:** 2023-06-13

**Authors:** Matthew Hellis, Suzanne Martin, Matthew Sheehan, Kevin Murphy

**Affiliations:** 1 Centre for Industrial and Engineering Optics, School of Physics, Clinical and Optometric Sciences, Technological University Dublin (TU Dublin), D07 ADY7, Dublin, Ireland; 2 FOCAS Research Institute, Technological University Dublin (TU Dublin), 13 Camden Row, D08 CKP1, Dublin, Ireland

## Abstract

Amblyopia is a significant issue for children worldwide, and current treatment methods have drawbacks that can hinder treatment effectiveness and/or patient experience. This study proposes a new treatment method using holographic diffusers while also comparing their optical characteristics to a current treatment method (Bangerter foils). Holographic diffusers were developed by optically patterning thin polymer layers on a micron scale. Two compositions of photopolymer (acrylamide and diacetone acrylamide based) are analysed herein. Characterisation shows that holographic diffusers of either composition can achieve a wide range of on-axis intensity reductions, allowing for precise and customisable treatment levels by altering recording exposure time in a low-cost and durable manner.

## 1. Introduction

Amblyopia is defined as a unilateral or bilateral reduction of the best-corrected visual acuity (BCVA) in the absence of organic ocular pathology [1–4]. The clinical definition for amblyopia is BCVA of 2 or more lines of acuity difference between eyes [3,4]. Additionally, diagnosis typically requires the presence of associated amblyogenic risk factors such as high refractive error. It is a significant impairment of normal visual development of children around the world, with prevalence of 1.9% in Australia [2], 4.2% in the UK [5], 1.1% in India [6], and 5.1% in Ireland [7]. An amblyopic person has a significantly increased risk of future issues ranging from reduced performance in school [8] to blindness [6].

Current treatment methods for non-strabismic amblyopia involve improving the visual stimulation to the amblyopic eye by correcting refractive error and/or obstruction such as ptosis. Persisting differences in BCVA then require adjunct methods to preferentially favour the amblyopic eye by temporarily penalising the sound eye with the use of; occlusion patching, atropine penalisation, or optical penalisation, such as with Bangerter foils [3,4]. Diffusive elements have previously been developed with the objective of treating amblyopia using a silica sol based diffuser [9].

The drawbacks of each treatment method can be significant, for patching compliance averaging less than 50% [3,10] and not addressing binocular fusion [4]; while for atropine side effects are common and include light sensitivity, ocular irritation, and headaches [3,4]. Bangerter foils have been proven inconsistent in achieving the mean acuity penalisation described by the filter label, as well as displaying potential differences between filters of the same labelled density [11–13]. Thus the Bangerter foil label should be interpreted as a nominal penalisation magnitude. The efficacy of Bangerter foils as a treatment for amblyopia was investigated by the Pediatric Eye Disease Investigation Group, and it found that the use of patching was not statistically superior to the use of Bangerter foils in their study (N=186) [14]. Optical testing of a range of Bangerter foils has previously been performed using a point spread function and modulation transfer function [13].

In addition to treating amblyopia, Bangerter foils have been shown to be useful in the management of intractable diplopia [15], and similarly, it is proposed that intractable diplopia may be managed with holographic diffusers.

Holography is a process by which the phase and intensity information of a wavefront is recorded within a medium, which can then be reconstructed [16]. Recording materials include silver halide, dichromated gelatin (DCG), thermoplastics, photoresists, and self-processing materials, including photopolymers [16]. Acrylamide-based holographic recording methods allow for the creation of mass-manufacturable elements that are cheap, lightweight, customisable, disposable, and robust [17]. Acrylamide-based holographic diffusers have previously been developed and refined using a single beam recording method [18,19]. While there are varied ways to record a diffuser holographically – such as a two-beam method [20] and contact copying [21] – neither of these methods allows control of the holograms diffusivity [18].

The toxicity of acrylamide monomers is a concern, as the World Health Organisation determined that acrylamide has a carcinogenic potency in rats [22]. It has been shown, however, that polyacrylamide has very low toxicity and is regularly used in the cosmetics industry [23]. The holographic recording process converts the acrylamide monomers into polyacrylamide. Given the proposed therapeutic use case here for the holographic diffusers, a further chemical composition of the photopolymer, which has been investigated using diacetone acrylamide [24] previously, is examined as a potential alternative to acrylamide. As previous work on the acrylamide holographic diffusers has been carried out [18,19], a more thorough investigation of the properties of the diacetone acrylamide holographic diffusers will be presented, including optimisation work.

The purpose of this study is to determine the optical characteristics of diacetone acrylamide photopolymer-based holographic diffusers and to compare and contrast their performance with acrylamide-based holographic diffusers and Bangerter foils. Section 2, details the composition of the photopolymers and the experimental methods behind analysing the diffusion efficiency and angular dependency [18], point spread function, modulation transfer function, and phase contrast microscopy [13]. Section 3 presents and discusses the experimental results, such as the optimisation and analysis of the diacetone acrylamide holographic diffusers. Then the above methods are used to compare the properties of the holographic diffusers (recorded in both compositions) and Bangerter foils. Finally, Section 4 highlights the conclusions from the study.

## 2. Methods

### 2.1 Photopolymer recording medium preparation

The preparation of the photopolymer involved creating a 10% w/vol Polyvinyl alcohol (PVA) solution (10.0 g of PVA) using deionised water (100 ml), which was the same preparation for diacetone acrylamide (DA) and acrylamide (AA) photopolymers. DA and AA (5.00g DA, 5.71g AA), bisacrylamide (1.00 g for DA, 1.14 g for AA), and triethanolamine (10.0 ml for DA and 11.4 ml for AA) was added to the PVA solution. Once thoroughly mixed, Erythrosine B dye was added with a concentration of 1.1 ${\mathrm{mg/cm}}^{3}$ .

Photopolymer was pipetted onto square polycarbonate substrates (LEXAN 8010) with an area of 1.9 ± 0.2 ${\mathrm{mm}}^{2}$ . The photopolymer was then spread to cover the sample uniformly and allowed to gravity settle for 24 hours before being covered with a 50 μ m thick cover layer (Melinex) to protect them from changes in humidity or other environmental factors. In addition, the cover layer prevents the development of surface features, thus ensuring that the recording occurs within the volume of the layer.

### 2.2 Holographic diffuser recording

**Fig. 1.**
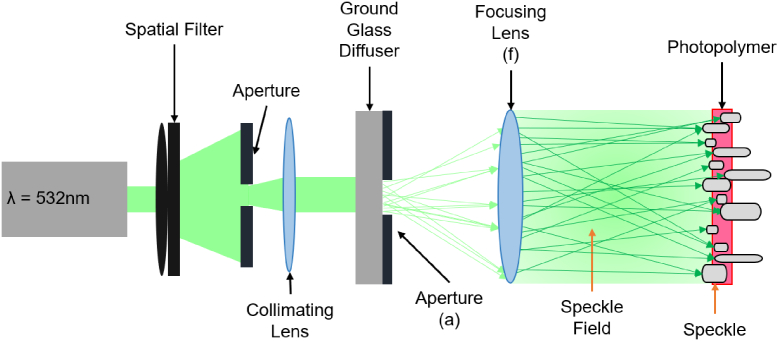
The setup used for the recording of holographic diffusers [18]. The aperture (a) and the focusing lens (f) are controlled to determine the minimum speckle sizing.

Diffusers were recorded using a single beam holographic method, as shown in Fig. 1. A laser beam with an output wavelength of 532 nm was passed through a spatial filter and then collimated. It was then propagated through a ground glass diffuser, a controllable aperture, and a focusing lens. The photopolymer sample is positioned in the back focal plane of the focusing lens, and the resulting speckle pattern is recorded within the volume of the photopolymer.

During the recording, the speckle size was determined by using Eq. (1), where a 12 mm aperture (a), a 150 mm focal length focusing lens (f), and a recording wavelength ( λ ) of 532 nm give a minimum speckle size ( σ ) of 16 μ m [18].

(1) $$\sigma =\frac{2.44\lambda f}{a}$$

The additional recording conditions are presented in Table 1, with exposure power at the sample and recording time varying throughout the experiment. The exposure power at the focal plane of the system ranged from 0.50-1.00 ${\mathrm{mW/cm}}^{2}$ ± 5% for the DA-based samples in steps of 0.1 ${\mathrm{mW/cm}}^{2}$ . Exposure power of 1.00 ${\mathrm{mW/cm}}^{2}$ ± 5% was used for the AA-based samples in line with previously reported findings [19]. The recording times used for DA diffusers ranged from 5-50 s, in steps of 5 s. For AA diffusers, the times varied from 25-125 s, in steps of 25 s.

**Table 1.** Recording conditions used to fabricate holographic diffusers in both diacetone acrylamide and acrylamide based photopolymer. Error on laser wavelength is <<1 nm with a linewidth <5 MHz.

Temperature (∘C)	18 ± 2
Relative Humidity (%)	40 ± 10
Aperture [a] (mm)	12.0 ± 0.5
Focal Length [f] (mm)	150 ± 2
Wavelength [ λ ] (nm)	532
Minimum Speckle Size ( μ m)	16.2 ± 0.7

Recorded diffusers were then UV bleached in a Dymax ECE 2000 Flood, removing any further polymerisation potential of any remaining monomers.

### 2.3 Diffusion efficiency

**Fig. 2.**
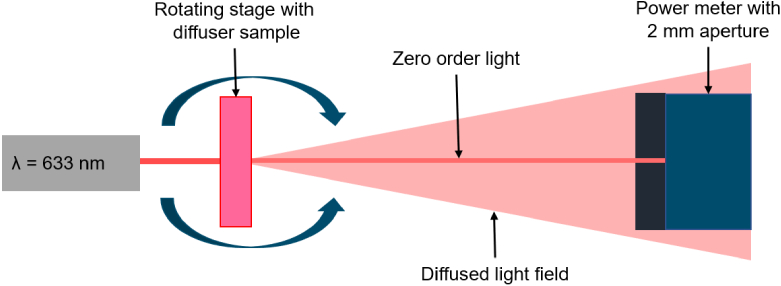
Experimental setup for the analysis of diffusion efficiency. The stage was rotated through a range of angles and the light incident on the power meter, covered by the 2 mm diameter aperture, was measured using a LabView program.

The optical setup for measuring diffusion efficiency is shown in Fig. 2. A holographic diffuser or Bangerter foil was placed in a rotating stage and illuminated by a laser beam ( λ=633 nm). The sample was swept through a range of angles and the on-axis intensity of the transmitted beam, passing through a 2 mm diameter aperture attached to the power meter housing, was measured at each angular position.

The diffusion efficiency (DE) of a sample is the proportion of the light diffused away from the optical axis by the diffuser. This is defined in Eq. (2) where $I_{s}$ is the intensity of the light with a sample present and $I_{c}$ is the intensity of the light with the control present [18]. The control sample was a bleached, unrecorded photopolymer sample of the same composition as the holograms. The control for the Bangerter foils was a piece of Lexan polycarbonate substrate, as this was the material that the Bangerter foils were adhered to for testing.

(2) $$DE=1-\frac{I_{s}}{I_{c}}$$

The DA-based samples and Bangerter foils were rotated through a $20^{\circ }$ range in increments of $0.5^{\circ }$ to analyse the diffusion efficiency. The AA-based samples used a range of $60^{\circ }$ in increments of $1^{\circ }$ .

It should be noted that the measurements from this technique were performed using a 633 nm probing beam. This was chosen to have comparability to previous work [13,18,19]. Initial experiments performed with a 532 nm probe beam show little or no differences at diffusion efficiencies > 95% and a higher diffusion efficiency found for probing in green in the region < 95%. In order to fully characterise this effect the diffusion efficiency will be assessed using a white light spectrometer in future work.

### 2.4 Measurement of point spread functions and modulation transfer functions

**Fig. 3.**
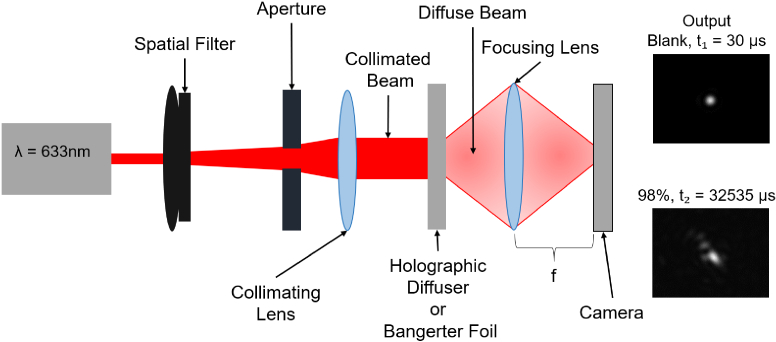
Setup for PSF measurement consisting of a laser, spatial filter, collimating lens, sample and focusing lens. An Alvium 1800 u-2050m camera was used to record the PSF in the focal plane of the focusing lens. Inset, two example PSFs for a blank sample with a 30 μ s integration time and a 98% DE holographic diffuser, 32535 μ s integration time.

The Point Spread Function (PSF) of each element under test was analysed using the setup shown in Fig. 3. A 633 nm laser beam was passed through a spatial filter to remove errant modes of the laser, and then collimated. The beam was incident normal to the surface of the diffuser/foil. A lens then focuses the beam on an Alvium 1800 u-2050m camera which was used to record the exposure. Exposure time was adjusted for each sample to ensure that the resultant PSF did not saturate the camera sensor.

The Modulation Transfer Function (MTF) was extracted from each PSF using self-created Python scripts. The code opens the PSF, finds the centre of the spot and averages grey levels through the radial profile while accounting for the exposure time required. A Fast Fourier Transform is then performed to determine and plot the MTF.

### 2.5 Phase contrast microscopy

Phase Contrast Microscopy (PCM) is a method of visualising transparent samples in which the phase change of the light traversing is converted to brightness variations. This was carried out using an Olympus BX51 phase contrast microscope with a DP72 camera using 40X magnification and 10X magnification (for Bangerter foils). Matlab scripts using autocorrelation code determined the speckle size from three phase contrast images taken at different points of the same holographic diffuser [18].

## 3. Results and discussion

### 3.1 Holographic recording optimisation for diacetone acrylamide-based photopolymer

It was necessary to determine the recording characteristics of the diacetone acrylamide (DA) photopolymer layers to optimise the performance of the holographic diffusers. While previous work has been completed in relation to optimising the recording of acrylamide (AA) based holographic diffusers [18,19], this study details the fundamental parameters and considerations for recording holographic diffusers with DA.

The DA photopolymer was prepared using a gravity settling technique, with 700 μ L of photopolymer achieving layers with thicknesses ranging from 50 to 130 μ m thick on the square format LEXAN polycarbonate substrate with an edge length of 4.3 cm. This range of thicknesses was significantly broader than anticipated, with potential causes of the variability being surface tension issues or the wettability of the polycarbonate substrate, with water on untreated glass having a contact angle of $55^{\circ }$ [25], and water on untreated polycarbonate having a contact angle of $84^{\circ }$ [26]. There is also scope for adding glycerol to the diacetone acrylamide photopolymer as this has been shown to improve layer stability and optical quality [27]. The acrylamide-based photopolymer attains high levels of consistency through roll-to-roll manufacturing, achieving rapid coating speeds of 18 m/min in lengths of tens of kilometres per roll [28]. Based on the robustness of roll-to-roll methods, it is anticipated that diacetone acrylamide uniformity would improve using such methods. Surface differences were noted between samples in the form of uneven layers. Thickness measurements were taken using a micrometre through the centre of the sample, corresponding with the measurement area for subsequent categorisation methods.

**Fig. 4.**
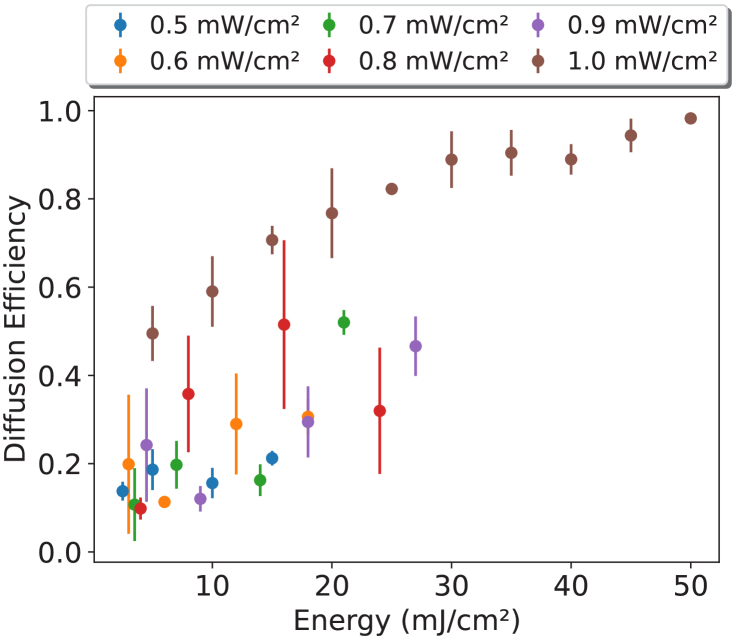
Diffusion Efficiency (DE) as a function of recording energy with the legend showing the recording power used to achieve the DE. Each data point is the mean of three diacetone acrylamide holographic diffusers. Error bars represent ± 1 standard deviation of the samples.

The recording of the DA holographic diffusers required the optimisation of parameters. The optimisation results are shown in Fig. 4 with a range of recording powers from 0.5 to 1 ${\mathrm{mW/cm}}^{2}$ analysed.

It was shown that the recordings with a power of 1 ${\mathrm{mW/cm}}^{2}$ achieved a diffusion efficiency of 99%, as well as producing a more predictable step between the energies delivered. In contrast to this, the next best performing recording powers obtained a diffusion efficiency of 50% for the 0.7, 0.8 ${\mathrm{mW/cm}}^{2}$ , with 0.9 ${\mathrm{mW/cm}}^{2}$ approaching 50%. This indicates that, like the acrylamide holographic diffusers [19], the diacetone acrylamide holographic diffusers record most reliably with 1 ${\mathrm{mW/cm}}^{2}$ of the tested intensities.

**Fig. 5.**
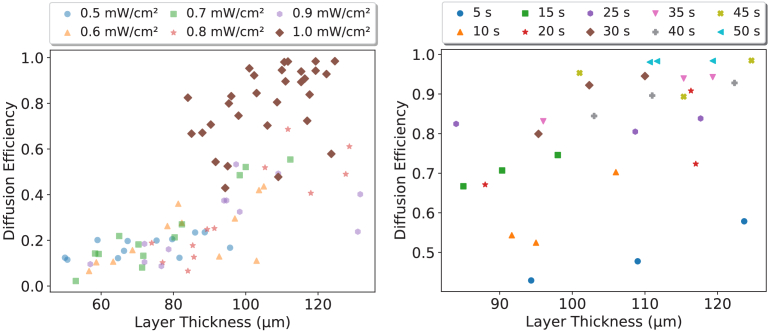
Diffusion Efficiency (DE) as a function of thickness. The relationship between recording power, thickness and diffusion efficiency for all recorded diffusers with the optimised 1 ${\mathrm{mW/cm}}^{2}$ highlighted (left) and the impact of time and thickness on diffusion efficiency for the 1 ${\mathrm{mW/cm}}^{2}$ recording power (right).

The range of diffusion efficiencies obtained for 1 ${\mathrm{mW/cm}}^{2}$ highlights the ability to produce diacetone acrylamide based holographic diffusers with diffusion efficiencies between 50-99%. These were obtained over a range of thicknesses from 85-125 μ m. Figure 5 show the diffusion efficiencies as a function of energy and thickness, respectively, and indicate a significant range of diffusion efficiencies can be developed.

By varying the recording power intensity and maintaining a thickness of 100 μ m, diffusers with diffusion efficiencies of between 10-99% are achievable. Diffusion efficiencies from 50-99% are achievable by keeping the recording power fixed at 1 ${\mathrm{mW/cm}}^{2}$ and varying the recording time. This broad range of possible diffusion efficiencies, attainable with the modification of a single recording parameter, is a significant advantage of both the AA and DA holographic diffusers for mass manufacturability.

**Fig. 6.**
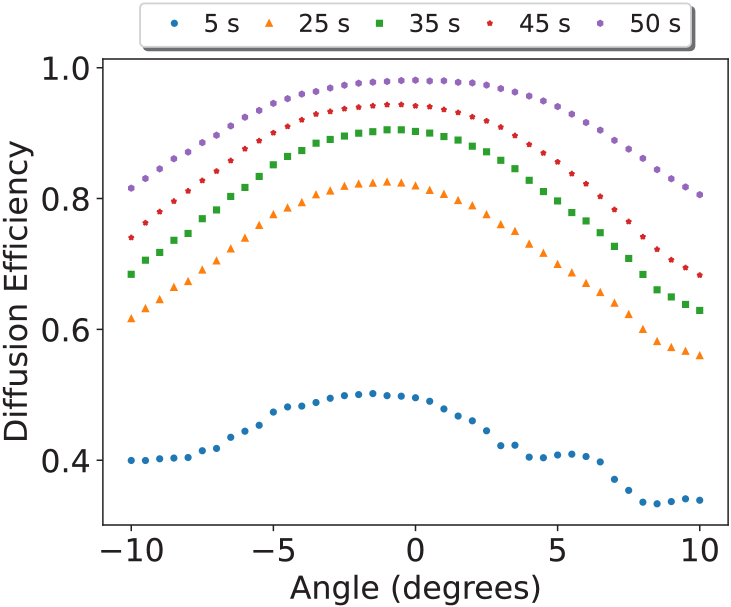
Diffusion Efficiency (DE) as a function of angle with the legend showing the recording time. A recording energy of 1 ${\mathrm{mW/cm}}^{2}$ was used to create these diffusers. Each dataset is the average of three datasets from different diffusers recorded under the same conditions with data points obtained every $0.5^{\circ }$ .

Figure 6 shows the relationship between the angle of incidence and the diffusion efficiency. It highlights that some angular dependence is evident in the DA holographic diffusers.

It was observed that the acceptance angle of the DA-based holographic diffusers broadened with increasing diffusion efficiency, with stronger diffusers exhibiting a $10^{\circ }$ range with a maximum reduction of 10% from peak diffusion efficiency.

This is of particular note in the use case of amblyopia treatment, as the patient would not be restricted to viewing at the zero order of the diffuser. While the central visual field spans $30^{\circ }$ [29], an investigation into suppression in amblyopes found that suppression was strongest in the central $10^{\circ }$ and extended throughout the $20^{\circ }$ tested [30]. As a gauge of comparison for what extent of the central field might be considered relevant, it is noteworthy that clinicians commonly test for metamorphopsia in the central $20^{\circ }$ field using the Amsler grid [31].

This indicates that while relatively wide acceptance angles have been presented for a holographic element, additional methods of controlling a patient’s ability to view obliquely may be required. Future work exploring multi-zone recording to introduce variable diffusion efficiency across selected regions of the hologram may defend against this use-case issue.

**Fig. 7.**
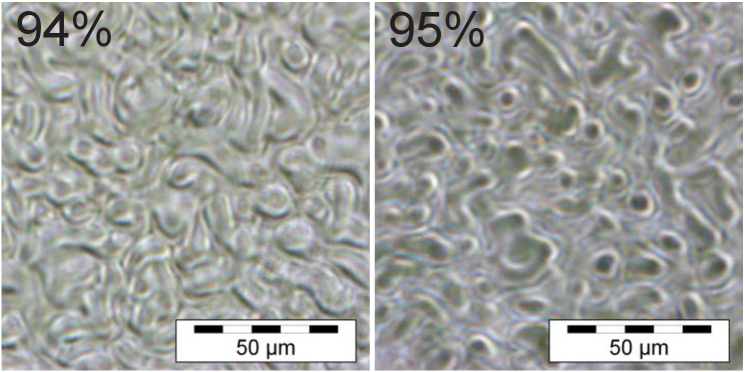
Phase contrast microscope image of the internal structure of holographic diffusers using diacetone acrylamide-based photopolymer.

The physical structure of the DA-based holographic diffusers features the recorded speckle pattern, which is controlled by the recording conditions as described in Eq. (2). Phase Contrast Microscopy (PCM) of the DA diffusers was carried out to observe the internal structure and confirm that no other process was developing within the sample, as shown in Fig. 7.

The PCM shows the formation of features where the speckle field developed within the three-dimensional space of the hologram. The speckle size for the samples composed of diacetone acrylamide photopolymer was determined to be 17.6 ± 2.5 μ m ( ± 1 SD). This agrees with the expected speckle size of 16.2 ± 0.7 μ m. It should be noted that the analysis method involves determining the size from the ${\mathrm{1/e}}^{2}$ points of the speckle [18].

The diacetone acrylamide-based photopolymer has demonstrated its versatility in producing holographic diffusers of a broad range of diffusion efficiencies that may be expected to be necessary in visual penalisation applications such as amblyopia therapy and in intractable diplopia.

### 3.2 Comparison of acrylamide-based holographic diffusers and Bangerter foils

In contrast with the diacetone acrylamide (DA) photopolymer layers, which had a range of 50-130 μ m with a mean thickness of 92 ± 21 μ m ( ± 1 SD) for 700 μ L of photopolymer, the acrylamide (AA) photopolymer layers with 700 μ L of photopolymer resulted in layers ranging from 48-75 μ m thick, with a mean thickness of 62 ± 7 μ m ( ± 1 SD). The acrylamide layers also displayed less variance across their surface than the diacetone acrylamide layers.

**Fig. 8.**
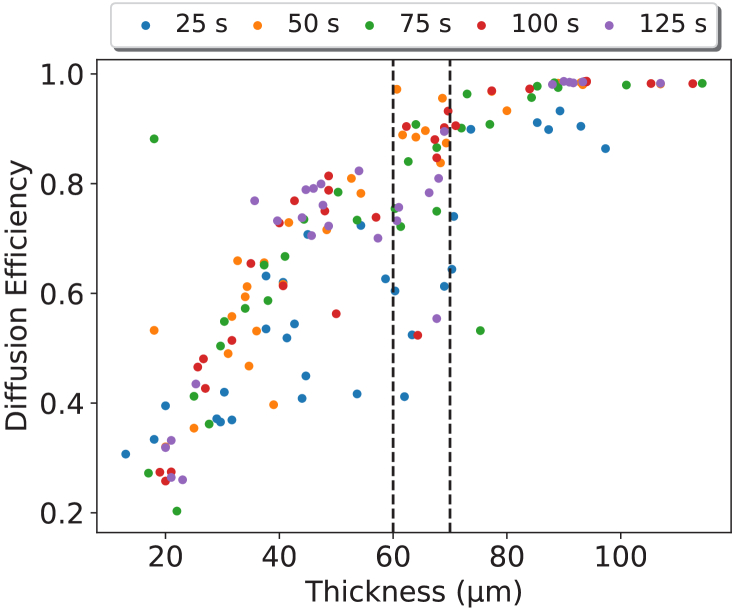
Diffusion Efficiency (DE) as a function of thickness with the legend showing the recording time used to achieve the DE. Recordings were obtained using a power of 1 ${\mathrm{mW/cm}}^{2}$ . The region enclosed by dashed black lines highlights a thickness range of 60-70 μ m, where diffusion efficiencies from 40-99% can be obtained by controlling the exposure time.

The acrylamide photopolymer layers were analysed according to a range of photopolymer volumes, thus providing a significant range of thicknesses for analysis as shown in Fig. 8, which shows the relationship between diffusion efficiency, thickness and recording time. A recording exposure power of 1 ${\mathrm{mW/cm}}^{2}$ was used for the AA-based holographic diffusers as the most reliable in producing holographic diffusers based on previous studies [19].

The thickness results in Fig. 8 demonstrate that high diffusion efficiencies are difficult to achieve for lower thicknesses (<30 μ m), whereas, for higher thicknesses (>80 μ m), all diffusers achieved very high diffusion efficiency. There is a thickness region where holographic diffusers can be produced with a DE ranging from 40-99% without changing the recording power of the laser or the thickness of the layer. This has the potential to be used for the manufacturing process at a large scale, allowing for a near-full range of production with the adjustment of a single parameter. This ability of both diacetone acrylamide and acrylamide-based photopolymer holographic diffusers to target customisable treatment levels based on diffusion efficiency and to taper treatment strength rather than penalisation time is a unique advantage over conventional treatment modalities.

**Fig. 9.**
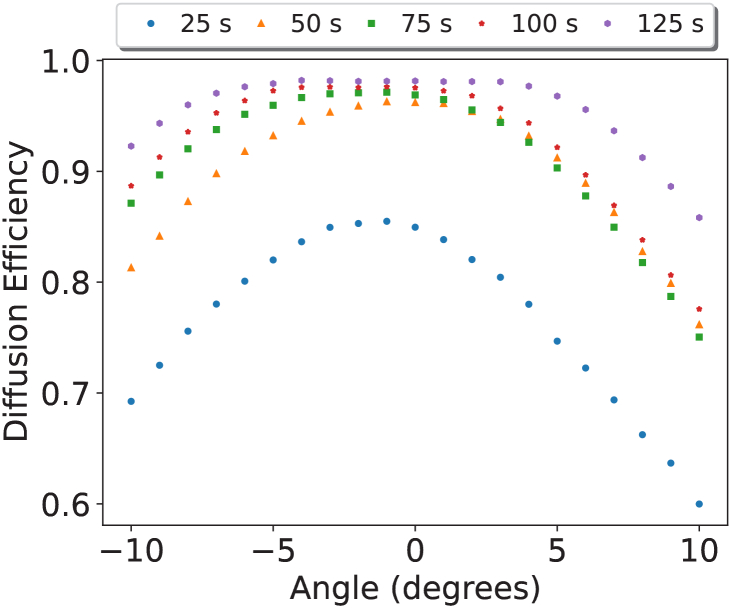
Diffusion Efficiency (DE) as a function of angle for a subset of acrylamide holographic diffusers. A recording power of 1 ${\mathrm{mW/cm}}^{2}$ was used, and each dataset is the average of three diffusers recorded for the time denoted in the legend. Data sampling was performed at $1^{\circ }$ increments.

Much like the diacetone acrylamide-based holographic diffusers, the acrylamide-based holographic diffusers show a large angular dependence for holographic devices. This is highlighted by comparing Fig. 6 and 9. The acceptance angles for the AA-based holographic diffusers widen as they record higher diffusion efficiencies.

**Fig. 10.**
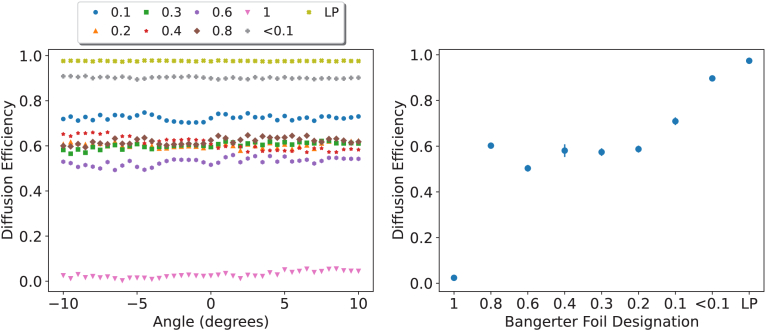
Diffusion Efficiency (DE) as a function of angle (left) and mean DE with standard deviation as the error (right) for all gradings of Bangerter foil.

Both AA and DA-based holographic diffusers demonstrate angular dependency, whereas Bangerter foils show no angular dependency. Figure 10, which shows diffusion efficiency related to the angle of incidence, displays no angular dependence for labelled gradings of Bangerter foils beyond sampling noise within the measurement technique.

The difference regarding angular dependence highlights that while the objective of Bangerter foils and holographic diffusers is the same, their methods of achieving it are significantly different. The lack of angular dependency is an advantage for Bangerter foils as a treatment method. Regarding holograms, multi-zone recording methods may be utilised to develop other angular dependencies. An example of such being an annulus centre of lower diffusion efficiency compared with a higher diffusion efficiency periphery, which may discourage patients from viewing obliquely through the holographic diffuser.

While both holographic options have the ability to fully customise the diffusion efficiency, the Bangerter foils demonstrated coarse diffusion efficiency increments, limiting the ability to discriminate and selectively tune this degradation parameter (Fig. 10). The foils 0.8 through 0.2 all penalise to roughly equal levels with regards to diffusion efficiency, with higher levels of diffusion efficiency attainable by the LP or <0.1 gradings. It has been previously reported that the optical properties of the 0.3 through 0.6 foils were similar with the 0.8 showing differences [13], notably, it has also been shown that in another study the 0.3 and 0.4 foils induced similar visual function deterioration [32]. Another study showed that the degree of visual degradation was not to the expected levels based on the labelling of the foils using near and distance optotype acuity, vernier acuity, and contrast sensitivity [11]. This collectively highlights issues with the grading of Bangerter foils and their clinical effect of graduated visual penalisation.

It is important to highlight that patching gives a singular treatment level of no vision in the penalised eye. By contrast, atropine penalisation may be considered to be a coarsely bi-modal treatment, i.e. distance viewing tasks of the child are not significantly penalised, but the near viewing tasks are. Notably, Bangerter foils have a greater ability for treatment-level customisation; however, holographic diffusers are potentially more tunable to patient needs.

**Fig. 11.**
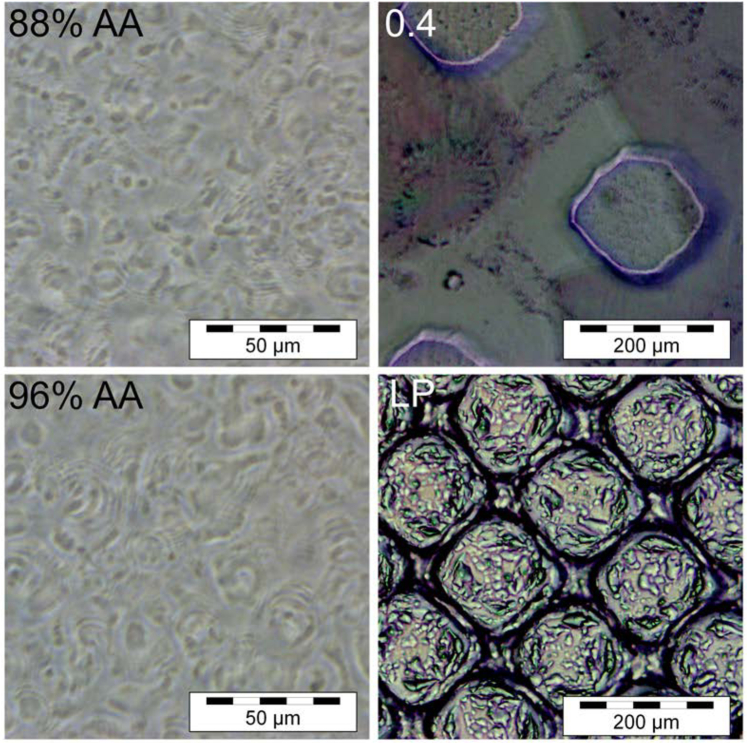
Examples obtained through phase contrast microscopy of the structure formed during the recording process for acrylamide-based holographic diffusers and the surface structure of a 0.4 and LP Bangerter foil

The structure of the acrylamide-based holographic diffuser is presented in Fig. 11, which shows the PCM images of AA-based holographic diffusers and Bangerter foils. The autocorrelation determination of the speckle size for the acrylamide photopolymer-based holographic diffusers was 15.2 ± 0.4 μ m. As with the DA speckles sizes (17.6 ± 2.5 μ m) this also agrees with the theoretical estimate of 16.2 ± 0.7 μ m. While the AA and DA-based holographic diffusers, in general, agree with regard to speckle size, the speckles recorded in DA-based diffusers tend to be slightly bigger and have more variability in size. This could be due to the larger monomer size of the DA or the differences in mass transport diffusion between the two compositions.

The features in the DA and AA-based holographic diffusers are all protected within the photopolymer layer, providing robustness against handling, and debris accumulation, in addition to environmental factors. This is a benefit that Bangerter foils do not possess, as the diffusing feature is imprinted on the surface and can thus be contaminated or damaged more easily. The structural difference highlights that while both holographic diffusers and Bangerter foils aim to penalise vision by inducing scatter, they do so with differing characteristics.

### 3.3 Analysis of point spread functions and modulation transfer functions

**Fig. 12.**
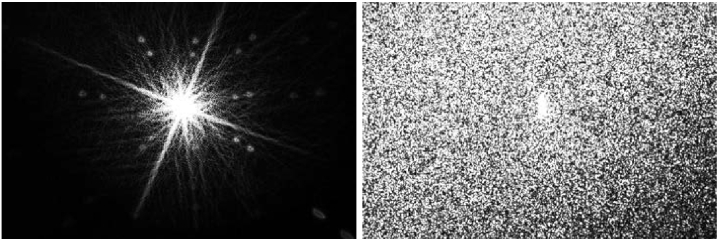
The point spread function of a blank sample (left) and 98% acrylamide holographic diffuser (right), both oversaturated to 36934 μ s integration time.

The comparative results, such as angular dependency and physical structure, highlight that the methods of achieving diffusion differ between the holographic diffusers and the Bangerter foils. The impact of the holographic diffuser is shown in the saturated profiles in Fig. 12.

**Fig. 13.**
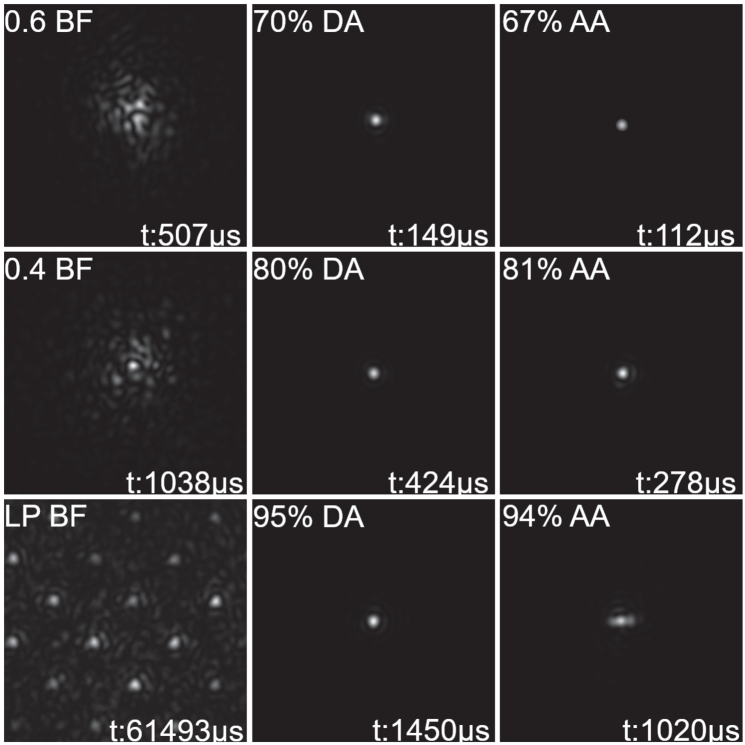
Point Spread Functions for the Bangerter foils (BF), diacetone acrylamide-based holographic diffusers (DA), and acrylamide-based holographic diffusers (AA).

The Point Spread Functions (PSFs), which are presented in Fig. 13 which highlights the PSF images collected show the difference in the behaviour of the Bangerter foils and holographic diffusers.

The Bangerter foils impart significant structure into the light beam. By contrast, holographic diffusers transmit the beam without artefacting the image. This lack of significant added structure highlights that any remaining vision maintained through the holographic diffuser has the potential to stimulate an element of binocularity for the patient. Despite the clean appearance of the PSF for the holographic diffusers, they do diffuse the light as demonstrated in Fig. 12, and ongoing pilot studies confirm that they do penalise both high and low contrast distance visual acuity. Figure 13 also highlights disparities observed in the exposure time required to collect the PSF images. This indicates that the samples performed at a higher level as they increased in diffusivity.

**Fig. 14.**
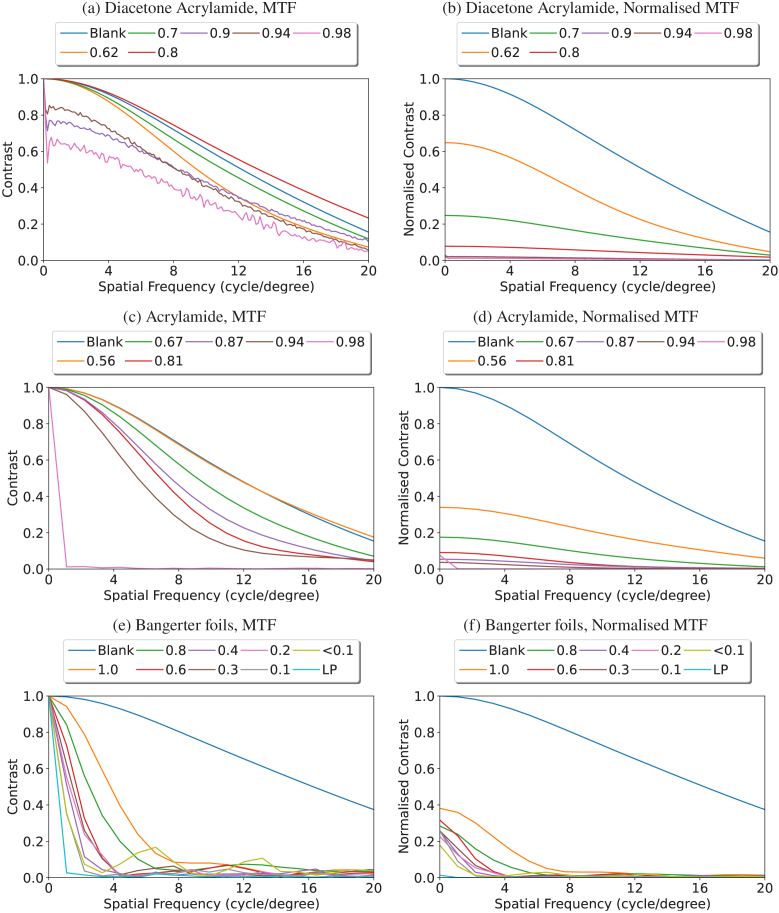
The Modulation Transfer Functions (MTFs) for the diffusers recorded in a diacetone acrylamide composition (a, b), acrylamide composition (c, d), and Bangerter foils (e, f). Graphs a, c, and e consist of each MTF starting at 1. This is shown to highlight the relative shapes of the MTFs compared to the blank. Graphs b, d, and f are normalised with the blank as the reference zero point, highlighting the impact of the element on the optical system.

The Modulation Transfer Functions (MTFs) of the elements measured in the system serve to determine the impact of the holographic diffuser or Bangerter foil on the light transmitted through it. The results of this analysis are shown in Fig. 14, which plots the MTF of DA and AA-based holographic diffusers and Bangerter foils.

In Figs. 14(a), and 14(c), the 0.8 and 0.56 diffuser strengths, respectively, are instances where the holographic diffuser artificially appears to be performing better than the control blank. This is due to the presentation of the data where each MTF starts at 1. This indicates that for those particular diffusers, the modulation of near-zero spatial frequencies (compared to the remainder of the frequency range) was proportionally greater compared to the same ratio for the blank sample.

For all instances, the DA gave a similarly shaped MTF, with significant noise developing on the >90% holographic diffusers. Below this threshold, the diffusers reduce the light transmitting through the diffuser, blurring the patient’s vision without introducing any further information. Conversely, above this threshold, as seen in both Fig. 14(a) and 14(b), the transmitted light is so reduced that a noise factor is present in the readings. Similar behaviour is demonstrated by the acrylamide holographic diffusers, with curves that follow similar paths and are reduced in magnitude, indicating the reduction of light without adding any structure. The MTFs demonstrate that for a diffusion efficiency >90%, the diffusers’ behaviour is more akin to occluders, with such a small amount of the spatial frequencies bypassing the holographic diffuser that they transmit very little useful spatial information.

The Bangerter foil modulation transfer function is of particular note, as all instances measured show that even for the weakest foil, the presence of the spatial frequencies is severely diminished, with frequencies above 7 cycles/degree for all foils and above 4 cycles/degree for the majority of foils being extinguished to the noise floor. This is a stark characterisation difference between the holographic diffusers and the Bangerter foils that supports the postulation that holographic diffusers may provide treatment novelties along with the benefit of stimulating some degree of binocularity.

As both Bangerter foils and high diffusion efficiency holographic diffusers can behave as occluders, this gives the ability for them to act akin to the patch in a treatment regime while retaining some stimulation to the sound eye.

## 4. Conclusion

Diacetone acrylamide-based photopolymer can be used to record holographic diffusers with diffusion efficiencies from 10-99%. This demonstrates that, despite less layer uniformity than the acrylamide-based samples, they allow for the same range of diffusion efficiencies as obtained for holographic diffusers recorded in the AA composition. The Bangerter foils, in contrast, have defined diffusion efficiency treatment levels of 5%, 60%, 90%, and 99%. Seemingly, they are more restrictive regarding the range of treatment parameters and options afforded. Holographic diffusers recorded in both diacetone acrylamide and acrylamide-based photopolymer permit a large range of spatial frequencies to be transmitted through but progressively reduce the magnitude of the spatial frequency components transmitted. This behaviour is unlike the Bangerter foils, which cut off the range of spatial frequencies transmitted before 7 cycles/degree for all instances. This is a significant difference, and it is therefore anticipated that the holographic diffusers will allow the stimulation of some binocularity during amblyopia treatment. Diacetone acrylamide is less toxic than acrylamide when in its monomer form and may be preferable to reduce risks in the creation of the diffusers, as similar diffusion efficiencies can be obtained with either formulation. This supports the potential use of holographic diffusers, recorded in either diacetone acrylamide or acrylamide-based photopolymer, as a treatment for amblyopia and intractable diplopia. Further work will determine the effect of the holographic diffusers on visual acuity.

## Data Availability

Data underlying the results presented in this paper are not publicly available at this time but may be obtained from the authors upon reasonable request.
